# Lack of skin sensitization hazard potential for alpha-glycosyl isoquercitrin (AGIQ) utilizing the Local Lymph Node Assay

**DOI:** 10.1016/j.toxrep.2022.05.021

**Published:** 2022-06-03

**Authors:** Puneet Vij, Douglas A. Donahue, Keith P. Burke, Shim-mo Hayashi, Robert R. Maronpot

**Affiliations:** a MB Research Laboratory, 1765 Wentz Road, Spinnerstown, PA 18968, USA; b Integrated Laboratory Systems, Inc., PO Box 13501, Research Triangle Park, NC 27709, USA; c Division of Food Additives, National Institute of Health Sciences, 3-25-26 Tonomachi, Kawasaki-ku, Kawasaki, Kanagawa 210-9501, Japan; d Maronpot Consulting LLC, 1612 Medfield Road, Raleigh, NC 27607, USA

**Keywords:** Alpha-glycosyl isoquercitrin, AGIQ, Isoquercitrin, Sensitization, Irritation, Local Lymph Node Assay, LLNA, Toxicity, Safety, CBA/J mice, Stimulation index (ices), OECD 442B

## Abstract

Skin sensitization is an important aspect of safety assessment and is a key component in the toxicological evaluation of chemicals. *alpha*-Glycosyl isoquercitrin (AGIQ), is marketed in Japan as a food additive and is generally recognized as safe (GRAS) by the expert panel of the Flavor and Extract Manufacturers Association (FEMA) in 2005 and the U.S. Food and Drug Administration (FDA) in 2007. The Local Lymph Node Assay (LLNA) was used to assess AGIQ’s potential to cause skin sensitization. Results indicate that no excessive irritation was observed after the irritation screen (ear swelling < 25 % and erythema score < 3) when AGIQ was tested at 5 %, 10 %, and 25 % in N, N-dimethyl formamide [DMF]. Based on lack of irritation, AGIQ was further evaluated at 10 %, 25 %, and 50 % in DMF in the main test resulting in stimulation indices of less than the positive threshold of 1.6 i.e., 1.2, 1.4, and 1.2 respectively. Therefore, AGIQ was not a dermal sensitizer in the LLNA.

## Highlights

• Topical application of AGIQ in DMF resulted in SI value less than 1.6.
• All animals survived in-life phase of the study and were observed to be normal.
• AGIQ is not a dermal sensitizer in the LLNA.
• AGIQ did not produce a SI value greater than 1.6 and an EC1.6 therefore could not be calculated.
• Concentration and homogeneity of AGIQ formulations were within acceptance criteria.

## Graphical Abstract

**Figure:**
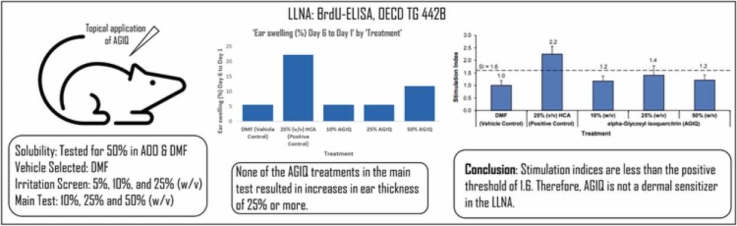

## 1 Introduction

Allergic disease is an important environmental and occupational health concern. Allergic contact dermatitis (ACD) is the second most reported occupational illness, accounting for 10–15 % of all occupational diseases. Skin sensitization remains a key endpoint in the toxicological evaluation of chemicals and their presence in commercial preparations [4]. Accordingly, methods used to identify skin sensitization hazard remains an important testing focus [6]. In late 1990s, the murine Local Lymph Node Assay (LLNA) was developed as an alternative in vivo test suitable for skin sensitization hazard identification and characterization, thereby fulfilling 3R (**R**eplace, **R**efine and **R**educe) criteria [10]. Among the animal tests, the LLNA has broad scientific and regulatory acceptance.

**Fig. 1:**
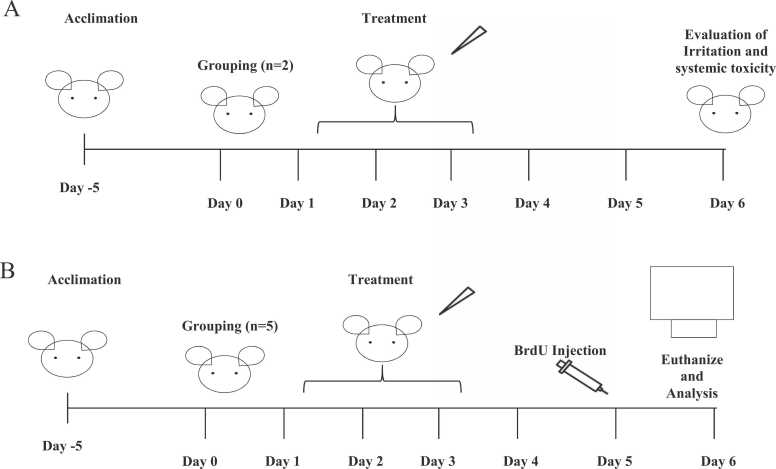
*Schematic diagram for the selection of study designs (Irritation Screen and Main Test).* (A) The Irritation screen and (B) Main test.

The basic principle underlying the traditional LLNA (OECD TG 429; [21]) is that chemical sensitizers induce a primary proliferation (induction phase) of lymphocytes in lymph nodes draining the application site, which can be quantified by measuring radiolabeled thymidine incorporation in the draining lymph node proliferating cells [14], [4]. The LLNA is the most common assay selected when necessary to measure the relative potency of an identified skin sensitizer, e.g ., in cosmetic industry safety assessment [6]. Modified protocols of the LLNA avoiding the use of a radiolabel for lymphocyte proliferation have been proposed including the 5-bromo-2-deoxyuridine (BrdU) based enzyme-linked immunosorbent assay (LLNA: BrdU-ELISA, [22]; See Fig. 1).

Low molecular weight (LMW) chemical sensitizers, referred to as haptens (or prohaptens), are too small to be allergenic and must bind to a protein to be allergenic. Three major cell types, keratinocytes, Langerhans cells, and T-lymphocytes, have been identified as central in the induction phase of ACD. The role of keratinocytes in both the induction and elicitation phases has been recently reviewed [2]. Haptens can directly stimulate keratinocytes present in the epidermis to release inflammatory mediators such as interleukins 1, 6 and 18, granulocyte-macrophage colony-stimulating factor, and tumor necrosis factor-α. The chemokine CCL2, which can recruit dendritic cells into the site of inflammation, is also upregulated in keratinocytes following hapten exposure. Langerhans cells (LCs), immature dendritic cells (DCs) present within the epidermis, take up and process haptenated protein within the major histocompatibility complex (MHC II). In the presence of the proper cytokine-signaling milieu, LCs migrate from the skin through the afferent lymphatics to lymph nodes draining the site of contact and become mature DCs during that process. DCs then present the haptenated peptide to responsive T-lymphocytes [13], [5]. Activated T-lymphocytes divide and differentiate into both T-effector and T-memory cells which starts the central phase of sensitization, and it is this allergen-driven proliferation response that is quantified in the LLNA [23].

**Fig. 2:**
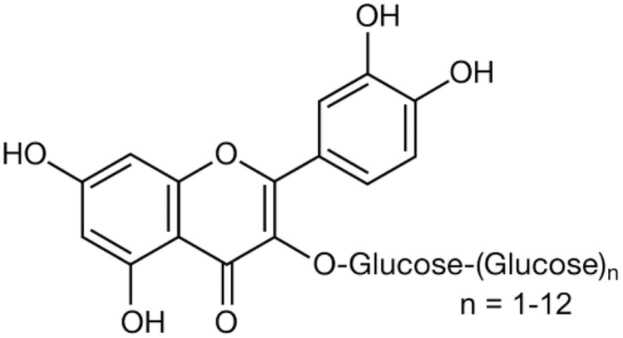
Alpha-glycosyl isoquercitrin.

AGIQ, also known as enzymatically modified isoquercitrin, is a polyphenolic flavonol glycoside derived by enzymatic glycosylation of rutin, which is contained in natural products such as citrus fruits, red beans, and buckwheat. AGIQ is a mixture of quercetin glycoside, consisting of isoquercitrin and its α-glucosylated derivatives of 1–10 or more of additional linear glucose moieties (Fig. 2). AGIQ is highly absorbable and has been shown to possess antioxidative properties. AGIQ was developed in 1987 and approved by the Japanese Ministry of Health and Welfare for use as a food additive in 1996. Isoquercitrin (quercetin-3-O-b-D-glucoside) is a rare natural compound, which has attracted much attention in the food and pharmaceutical industries due to its long list of beneficial properties [26]. Based on its favorable safety profile, AGIQ has been concluded by the Expert Panel of the Flavor and Extract Manufacturers Association (FEMA) (FEMA No. 4225) as a Generally Recognized As Safe (GRAS) compound in 2005 [24]. The U.S. Food and Drug Administration (US FDA) has granted a GRAS status for AGIQ as an antioxidant as well, based on the details given in the GRAS Notice (GRN 000220) [7].

## 2 Materials and methods

### 2.1 Animal husbandry and maintenance

Female CBA/J mice, seven weeks old, in good health were ordered from Jackson Laboratories (Bar Harbor, ME). Thirty-one nulliparous and non-pregnant female mice were randomly assigned to the test groups for the preliminary dermal irritation screen and the main test following an acclimation period of at least five days. At the start of the study, these mice were eight weeks old. The weight variation of the animals at study start did not exceed ± 20 % of the mean body weight in each study phase. The animals were examined to ensure that no skin lesions were present on the ears prior to start of study. Certified PMI Rodent Chow (Diet no. 5002, Animal Specialties and Provisions, Quakertown, PA) and water were available ad libitum. A cleaned vermin-free animal room, reserved exclusively for this study, was temperature-controlled and maintained with a 12-hour light and dark cycle. Temperature and humidity were continuously monitored using automatic recording devices at the AAALAC International accredited MB Research Labs facility. All procedures and protocols were reviewed and approved by the MB Research Labs Institutional Animal Care and Use Committee (IACUC) and performed in accordance with the guidelines published by the OECD.

### 2.2 Chemicals

Acetone: Olive oil, AOO 4:1 [acetone (purity: 99 %, Fisher Scientific, Hampton, NH) and olive oil (Sigma-Aldrich, St. Louis, MO)] and N, N- Dimethylformamide, DMF (Acros Organics) were used as vehicles in the preliminary solubility test. Based on solubility results, DMF was selected as the vehicle. Ammonium chloride (NH_4_Cl, MW: 53.491 g/mol), potassium bicarbonate (NaHCO_3_, 100.115 g/mol) and ethylene-diamine-tetraacetic acid (EDTA) were purchased from Fisher Scientific (Hampton, NH). Fetal Bovine Serum (FBS) was purchased from Serum Source International (Charlotte, NC). α-hexylcinnamaldehyde (HCA, 85 % technical grade) was purchased from Sigma-Aldrich (Saint Louis, MO). A BrdU-specific colorimetric cell proliferation Enzyme-Linked Immunosorbent Assay (ELISA) kit, Lot no. 34570800, manufactured by Roche Applied Science (Indianapolis, IN) was obtained from Sigma-Aldrich (Saint Louis, MO).

### 2.3 Solubility testing

AGIQ was tested for solubility at 50 % (w/v) in AOO and at 50 % (w/v) in DMF. A solution of AGIQ in AOO could not be made, but a solution was obtained in DMF. AGIQ formulations (50 % and 0.1 % in DMF) were prepared. Three samples were taken from each AGIQ formulation (from the top, middle, and bottom) and sent to the Integrated Laboratory Systems, Inc. for dose formulation analysis and stability assessment (see Section 2.4). The samples were analyzed in duplicate for concentration and homogeneity. The analysis found that both concentration and homogeneity were within acceptable ranges; ≤ 15 % of the target concentrations. AGIQ in DMF was found to be stable for at least 24 h at room temperature and for 10 days at 2–8 °C. A vehicle control DMF sample was considered as a blank for the analysis.

### 2.4 Analytical method for validation and stability check

Determination of AGIQ concentration levels in dose formulation samples was accomplished using ultra-high-performance liquid chromatography (UHPLC/UV) by using a validated analytical method. The diluent used for the validation and subsequent analysis was 0.1 % Phosphoric acid (H_3_PO_4_) in distilled water: Acetonitrile (C_2_H_3_N) (82:18) (v/v). The samples were analyzed by reverse-phase chromatography at 35 °C column temperature, using an Agilent Zorbax Eclipse C18 (50 × 2.1 mm, 1.8 µm) under isocratic elution conditions. The mobile phases consisted of 0.1 % H_3_PO_4_ in distilled water as mobile phase A (MPA) and C_2_H_3_N as mobile phase B (MPB) at a flow rate of 0.35 mL/min and 84:16 (MPA: MPB). The effluent was analyzed at 254 nm by a photo diode array detector. The calibration curve for both analyses was fit using a linear regression analysis of the sum of the area of the AGIQ peaks (~ 0.6–2 min) versus the nominal AGIQ concentration. The six standards have an analytical range of 10–60 μg/mL. Two separate concentration verification (CV) analyses were performed. The standard curves prepared for each analytical analysis demonstrated results within acceptance criteria for each analysis (data not shown).

### 2.5 Dosing

Treatment was performed via topical application of AGIQ concentrations to the dorsum of each ear once daily for three consecutive days. The formulations were mixed by vortex before each dose. The AGIQ formulations were spread over the entire dorsal surface of the ear (25 μl/ear).

#### 2.5.1 Irritation screen

After the stability analysis, the AGIQ formulations of 5 %, 10 % and 25 % (w/v) in DMF were prepared and three samples were taken from each formulation (from the top, middle, and bottom) and analyzed for concentration (results within acceptable ranges, data not shown). Three groups of CBA/J mice (two animals per group) were dosed with increasing concentrations of AGIQ i.e., 5 %, 10 % and 25 % (w/v) in DMF.

#### 2.5.2 Main test

The AGIQ concentrations used in the main LLNA study were 10 %, 25 %, and 50 % (w/v) in DMF such that the maximum concentration tested avoided both overt systemic toxicity and excessive local dermal irritation. Like the irritation screen, three samples were taken from each formulation (from the top, middle, and bottom) and sent for concentration analysis. The results were within acceptable ranges (data not shown). The moderate sensitizer (and irritant) 25 % HCA in DMF was used as a positive control. Five groups of CBA/J mice (five animals per group) were treated by topical application of the AGIQ concentrations, vehicle control, or positive control in the same manner as in the screen. All animals were observed once daily throughout the study for clinical signs, either of local irritation at the application site or systemic toxicity, and for mortality.

### 2.6 Body weight and ear measurements

Body weights were recorded on day one immediately prior to dosing and on day six (prior to euthanasia). Ear thickness measurements were performed on day one prior to dosing, on day three before the third AGIQ/vehicle/positive control dosing (approximately 48 h after the first AGIQ application), and on day six before euthanasia (approximately 120 h after the first dose) using a calibrated digital micrometer. Changes in ear thickness on day three and day six relative to day one were expressed as a percent of the day one pre-dose values.

### 2.7 Lymph node isolation and processing

#### 2.7.1 Irritation screen

On day six of the dermal irritation screen, each mouse was euthanized using carbon dioxide (CO_2_) _+_ asphyxiation, followed by exsanguination via the jugular vein. Gross observations of the auricular lymph nodes were made; the lymph nodes were not collected.

#### 2.7.2 Main test

On day five of the main test, approximately 96 h following the initial dose and approximately 24 h prior to euthanasia, the mice were injected with BrdU (10 mg/mL) in Dulbecco's Phosphate-Buffered Saline (dPBS) at a dose of 500 μl per mouse. The BrdU solution was administered by intraperitoneal injection using a 26-gauge needle. As a thymidine analog, BrdU becomes incorporated into the DNA of proliferating cells, including proliferating nodal lymphocytes. On day six, each mouse was euthanized using CO_2_ asphyxiation followed by exsanguination via the jugular vein. Gross observations of the auricular lymph nodes were made, and the lymph nodes were collected. The auricular lymph nodes (left and right sides of individual mice) were combined for each animal into a 1.5 mL RNase free microtube containing 100 μl FBS while keeping the microtubes on ice. These lymph nodes were disrupted using a pestle to break open the lymph node capsule to release single lymph node cells (LNCs) to create a single cell suspension. Cells remaining on the pestle were rinsed into the microtube using 1000 μl of a 1X ammonium chloride red blood cell lysis solution (ammonium chloride (NH_4_Cl), potassium bicarbonate KHCO_3_ and ethylenediamine tetraacetic acid (EDTA) and Dulbecco’s Phosphate- Buffered Saline (dPBS). LNCs were incubated on ice in the lysis solution for approximately 10 min. After incubation, LNCs were centrifuged at 1000 rpm for approximately five minutes at 4 °C. After centrifugation, 1000 μl of the supernatant was removed and 1000 μl of ice-cold dPBS was added to the LNCs into individual microtubes and vortexed. This step was repeated to remove the excess lysis solution from LNCs. Then, LNCs were resuspended in 900 μl of ice-cold RPMI-1640 storage medium supplemented with 10 % FBS and 1.0 % penicillin-streptomycin and stored in the refrigerator (2–8 °C) overnight.

### 2.8 Determination of BrdU Incorporation

The BrdU kit measured the BrdU incorporation in the lymph node cell (LNC) suspensions by ELISA using a commercial kit (e.g., Roche Applied Science, Indianapolis, IN, USA, cat. no. 11647229001). The study followed the kit instructions and GLP-validated MB Research standard operating procedures (SOPs). Briefly, from each of the single-cell LNC suspensions, 100 μl LNC suspension was brought to a total volume of 500 μl with Hanks' Balanced Salt Solution (HBSS) and vortexed. A volume of 100 μl of the LNC suspension was added to the wells of a 96 well flat-bottom microplate, in triplicate. The cells were dried in an oven at 60 °C, then denatured and fixed using the buffer solution supplied with the ELISA kit. After denaturation and fixation of the LNC, anti-BrdU antibody was added to each well and allowed to react. Excess unbound anti-BrdU antibody solution was then removed by multiple washing steps and the substrate solution was added and allowed to generate chromogen. Absorbance was measured using a MicroQuant MQX200 plate reader (Bio-Tek Instruments, Winooski, VT), at 370 nm with a reference wavelength of 492 nm. The mean optical density (OD) values of dPBS treated “blank” wells were used as a reference.

### 2.9 Analysis of stimulation index data and EC1.6 calculation

For each animal, lymph node cell proliferation was determined by BrdU-ELISA and the mean optical density (OD), and standard deviation (S.D.) were then calculated for each group. The mean OD for each animal was divided by the mean of proliferating lymphocytes in the vehicle control group. This “Test/Control Ratio” is the “Stimulation Index” (SI) and was calculated for each animal as follows:

**SI per animal = Individual Animal Mean OD _Test Substance_/Group Mean OD _Vehicle_**

When concentrations yield SI values both above and below 1.6, in an increasing linear dose response, the concentration at which SI = 1.6 (EC1.6) can be calculated according to the following:

**EC1.6 = c + (1.6 − d) (b − d) × (a − c)**

where: a = test article concentration at SI value “b”.

   b = SI value nearest to but greater than 1.6.

   c = test article concentration at SI value “d”.

   d = SI value nearest to but less than 1.6.

The AGIQ EC1.6 could not be calculated in this study because no concentration of AGIQ produced an SI value greater than 1.6.

### 2.10 Statistical analysis

For each AGIQ-treated group, the individual animal SI values along with the mean group SI and standard deviation were calculated, and ANOVA followed by the Student-Newman-Keul’s test, using lnStat™ (version 3.06, 32 bit for Windows; GraphPad Software, San Diego, CA). was performed to statistically compare each AGIQ dose group to the vehicle control group. Although specified in the test guidelines, these calculations and results were not incorporated into the interpretation of the data. A SI value of 1.6 or more is the sole determinant for a positive sensitization response.

## 3 Results

### 3.1 Mortality and clinical observations

After application of AGIQ at concentrations of 10 %, 25 %, and 50 % in DMF, no abnormal clinical observations were present. No mortality was observed on the study.

### 3.2 Ear thickness as a measure of dermal irritation

During the irritation screen, none of the AGIQ treatments resulted in increases in ear thickness of 25 % or more, and therefore AGIQ was not considered irritating at these concentrations. Based on these screen results see Table 1 for scoring criteria (Table 1) and AGIQ solubility data, AGIQ concentrations of 10 %, 25 %, and 50 % (w/v) in DMF, were chosen for the main assay. See Table 4, Table 5 for irritation screen and main test ear thickness measurements results. OECD guideline 442B states that an increase in ear thickness exceeding the threshold value of 25 % is considered to be indicative for excessive local skin irritation and this threshold was not exceeded in any AGIQ treated group.

**Table 1:** Ear thickening scoring criteria.

Observation	Score
No erythema	0
Very slight erythema (barely perceptible)	1
Well-defined erythema	2
Moderate to severe erythema	3
Severe erythema (beet redness) to eschar formation preventing grading of erythema	4

**Table 2:** Irritation screen body weight measurements for AGIQ concentrations of 5 %, 10 %, and 25 % (n = 2 per group).

Irritation screen: body weights (g)						
Treatment	Mouse ID	Day 1	Day 6	Weight Change (g)	Mean weights	
					Day 1	Day 6
5 % AGIQ in DMF	**1**	18.8	20.8	2.0	19.5	21.3
	**2**	20.2	21.7	1.5		
10 % AGIQ in DMF	**3**	21.1	21.5	0.4	20.9	21.8
	**4**	20.7	22.0	1.3		
25 % AGIQ in DMF	**5**	20.5	22.7	2.2	21.4	22.5
	**6**	22.3	22.3	0.0

**Table 3:** Main test body weight measurements for vehicle, positive control and AGIQ concentrations of 10 %, 25 % and 50 % (n = 5 per group).

Main test: body weights (g)				
Treatment	Mouse ID	Mean weights		Mean weight change
		Day 1	Day 6	
N, N-Dimethylformamide (DMF)	7–11	23.4	24.3	0.8
25 % (v/v) HCA (Positive Control in DMF)	12–16	20.5	21.4	0.9
10 % AGIQ in DMF	17–21	22.9	23.2	0.4
25 % AGIQ in DMF	22–26	24.4	24.7	0.4
50 % (w/v) AGIQ in DMF	27–31	21.2	22.3	1.1

**Table 4:** Irritation screen ear thickness measurements for 5 %, 10 %, and 25 % (n = 2 per group).

Irritation screen: ear thickness (mm)					
Treatment	Mean ear thickness			% Ear swelling	
	Day 1	Day 3	Day 6	Day 3–1	Day 6–1
5 % AGIQ in DMF	0.17	0.17	0.18	0.0	5.9
10 % AGIQ in DMF	0.18	0.19	0.17	5.6	-5.6
25 % AGIQ in DMF	0.19	0.19	0.18	0.0	-5.3

**Table 5:** Main test ear thickness measurements for vehicle, positive control and AGIQ concentrations of 10 %, 25 %, and 50 % (n = 5 per group).

Main test: ear thickness (mm)					
Treatment	Mean ear thickness			% Ear swelling	
	Day 1	Day 3	Day 6	Day 3–1	Day 6–1
N, N-Dimethylformamide (DMF)	0.18	0.18	0.19	0.0	5.6
25 % (v/v) HCA (Positive Control in DMF)	0.18	0.20	0.22	11.1	22.2
10 % AGIQ in DMF	0.18	0.19	0.19	5.6	5.6
25 % AGIQ in DMF	0.18	0.18	0.19	0.0	5.6
50 % (w/v) AGIQ in DMF	0.17	0.18	0.19	5.9	11.8

### 3.3 Body weights, mortality and systemic observations and erythema scores

In the main study, slight changes in body weights were observed but occurred in a non-dose-dependent manner and were not considered treatment-related (See Tables 2-3 for irritation screen and main test body weight changes). On day one, body weight for the dermal irritation screen animals and the main test animals ranged from 18.8 to 22.3 g (g) and 18.1 to 25.6 g respectively. All animals survived the in-life phase of the study and were observed to be normal (SeeTable 6). Prior to the study start, all animals were observed to ensure that no skin lesions were present on the ears and appeared normal. None of the AGIQ treatments in the irritation screen produced erythema. In the main test, very slight to no erythema was noted for the 50 % AGIQ treatment group.

**Table 6:** Clinical observations, body weight observations and lymph node size for vehicle, positive control and AGIQ concentrations of 10 %, 25 %, and 50 % (n = 5 per group).

Treatment	Systemic observations/body weights	Lymph node size
N, N-Dimethylformamide (DMF)	No abnormal physical signs were observed. All animals gained weight by study termination.	Small
25 % (v/v) HCA (Positive Control in DMF)	No abnormal physical signs were observed. All animals gained weight by study termination.	Medium-Large
10 % AGIQ in DMF	No abnormal physical signs were observed. Two animals gained and three animals lost weight by study termination.	Small
25 % AGIQ in DMF	No abnormal physical signs were observed. Four animals gained and one animal lost weight by study termination.	Small
50 % (w/v) AGIQ in DMF	No abnormal physical signs were observed. All animals gained weight by study termination.	Small

### 3.4 Stimulation index (SI)

Stimulation indices (SI) were calculated by dividing the mean optical density value (a representation of the total amount of LNC proliferation) for each animal by the mean optical density value for the vehicle control group (see Table 7). The mean SI and standard deviation (SD) for each treatment group was then calculated. A result is regarded as a positive response in the LLNA if at least one concentration results in a 1.6-fold or greater increase in LNC proliferation relative to that of vehicle control animals, as indicated by a SI of 1.6 or more. Although two of five animals in the 25 % AGIQ-treated group could be considered borderline positive, the group mean SI did not reach the 1.6 cut-off, the group results were not statistically different from the vehicle control, and there was no dose-dependent increase. A test substance is regarded as a sensitizer in the LLNA, if exposure to one or more test substance concentrations results in a 1.6-fold or greater increase in incorporation of BrdU compared with concurrent controls, as indicated by the SI. The SI obtained with AGIQ concentrations up to 50 % in the present study were below 1.6 and, therefore, support non-sensitizing properties of AGIQ when tested in a DMF vehicle.

**Table 7:** Stimulation indices (S.I) of vehicle, positive control and all the AGIQ treatment groups.

Treatment	Vehicle	Stimulation Index (SI)
N, N-Dimethylformamide (DMF)	None	**1.0**
25 % (v/v) HCA (Positive Control in DMF)	DMF	**2.2**^a^
10 % AGIQ in DMF	DMF	**1.2**
25 % AGIQ in DMF	DMF	**1.4**
50 % (w/v) AGIQ in DMF	DMF	**1.2**

a SI value of greater than 1.6.

## 4 Discussion

To support expanded global marketing of food products containing AGIQ, comprehensive testing of skin sensitization potential of AGIQ was conducted by using GLP complaint LLNA-ELISA according to current regulatory LLNA test guidelines. The LLNA: BrdU-ELISA has been reviewed, validated, and approved for use internationally, and its performance is regarded as equivalent to the traditional LLNA. Lehmann ($year$) [15]. The LLNA is an alternative approach to traditional guinea pig methods and in comparison, provides important animal welfare benefits by utilization of the 3R principles. The LLNA: BrdU-ELISA assay has recently been used for the proposed GHS sub-categorization criteria on the skin sensitization potency category of chemicals [17] and is currently under consideration. The LLNA: BrdU-ELISA test was also used for the validation of BALB/c mice to be used in this assay [9]. The present study fills a critical information gap regarding potential dermal sensitization response of AGIQ. In this study, the ex vivo LLNA: BrdU-ELISA test was used to distinguish the sensitizing and irritation potential of AGIQ in two end points: lymphocyte proliferation and ear swelling. A similar study was performed by Moon et al. [19] demonstrated the skin-sensitizing potential of Asarum radix oil using the LLNA: BrdU-ELISA test at concentrations of 10 %, 25 %, and 50 % (v/v).

Determination of the contact sensitization potential of a chemical is an important component of the safety assessment process and the murine Local Lymph Node Assay (LLNA) has emerged as the preferred assay for this evaluation. This animal-based (Female CBA/J mice) assay seeks to identify contact allergens as a function of events induced during the acquisition of skin sensitization, more specifically, lymphocyte proliferative responses induced in the regional lymph nodes of mice exposed topically to test chemicals.

AGIQ is a mixture of isoquercitrins with one or more added glucose moieties [1]. Isoquercitrin properties includes anti-inflammatory, hypotensive, anti-mutagenesis, anti-oxidative, anti-depressant, hypolipidemic and anti-viral effects [11], [12], [16], [25], [3]. Results from recent studies examining the toxicological potential of AGIQ include lack of in vivo genotoxicity [8], lack of toxicity in 90-day rat studies at dietary doses up to 5 % [20], and lack of in vivo carcinogenicity in rasH2 mice [18]. These studies along with the present findings of lack of sensitizing potential support the safety of current use levels of AGIQ in food and beverages.

Based on analytical analysis of the dosing formulations, the test substance was homogeneous, percent recoveries were within acceptance criteria prior to dose administration, and dose formulation concentrations of AGIQ in DMF were verified. Aside from minimal changes in body weight, study animals did not show any signs of toxicity or any mortality, ear thickness was less than 25 % in treated mice, and auricular lymph node cell counts were not increased after application of AGIQ. Based on study findings, AGIQ was negative in the LLNA when tested up to 50 % (maximum concentration tested) and is classified as a non-sensitizer.

## Funding

This study was funded by San-Ei Gen F.F.I., Inc., Osaka, Japan.

## Author Statement

I have addressed the two comments requested by the reviewers.

## Declaration of Competing Interest

The authors declare that they have no known competing financial interests or personal relationships that could have appeared to influence the work reported in this paper.
